# A systematic review and meta-analysis of the morbidity of the donor-site of flaps harvested based on the first intermetatarsal artery^✰^

**DOI:** 10.1016/j.jpra.2026.01.009

**Published:** 2026-01-17

**Authors:** Virginia E Knutson, Giorgio Pajardi, Giulia Del Vecchio, Macarena Vizcay, Stephanie Hili, Francesco Zanchetta, Luigi Troisi

**Affiliations:** a Maastricht University Faculty of Health, Medicine and Life Sciences, Maastricht 6229 ER, the Netherlands; b Reconstructive Microsurgery Service, MultiMedica Group, Milan 20123, Italy; c School of Specialization in Plastic, Reconstructive and Aesthetic Surgery, Milan University, Milan 20122, Italy

**Keywords:** Meta-analysis, Donor-site morbidity, Microsurgery, Free toe flap, Toe transfer, Dorsal pedis flap

## Abstract

**Background:**

Free tissue flaps from the foot have become an increasingly reliable tool in modern reconstructive surgery; however, the field lacks an empirical analysis focused on donor-site morbidity. This systematic review and meta-analysis aimed to evaluate the incidence of donor-site morbidity following dorsalis pedis and free toe flap harvest.

**Methods:**

This systematic review and meta-analysis was performed according to the PRISMA guidelines. A systematic search of PubMed, Embase, CENTRAL, and clinicaltrials.gov was performed on March 3, 2025, to identify eligible studies involving adults who underwent free flap harvest from the toes or dorsum of the foot and reported postoperative donor-site morbidity outcomes. Eligible designs included case series (≥5 patients), observational studies, randomized controlled trials, and controlled clinical trials published in English. Letters, reviews, editorials, conference abstracts, and animal and cadaveric studies were excluded. Two reviewers independently screened studies, extracted data, and assessed bias.

**Results:**

We included 394 cases of free flap harvest (three studies on dorsalis pedis flaps and 11 on toe flaps) across 14 studies published between 2000 and 2025. The percentage of patients experiencing objective and subjective donor-site morbidity was 2.99% and 3.32% respectively. Wound dehiscence, delayed healing, and hematoma were the most common objective complications, while daily life disturbance, pain, and numbness occurred most frequently in the subjective morbidity domain.

**Conclusions:**

The occurrence of donor-site morbidity is relatively low, although substantial heterogeneity limits the strength of these results. This review offers comprehensive insights into donor-site morbidity of foot free flaps, aiming to improve patient and physician awareness.

## Introduction

Free flap procedures have become an increasingly reliable tool in modern reconstructive surgery, offering reconstructive microsurgeons a variety of donor-sites suited to diverse anatomical and functional needs. The foot offers unique advantages as a donor-site, particularly because it is a source of specialized tissue types, including digits and their components.^1^^,^^2^ Among the various foot-originating free flaps, two free flaps—the dorsalis pedis flap and free toe flap- are commonly used due to their physical characteristics and practical location. The free toe flap, often utilized in finger reconstruction owing to its compositional and visual similarities, has multiple variants, including the trimmed great toe, toe pulp, and wraparound flaps. Selecting the appropriate flap requires thorough presurgical planning, weighing the suitability of the flap for the recipient site against the risk of complications, including donor-site morbidity. Donor-site morbidity may commonly arise as mild, temporary postoperative deficits or minor aesthetic concerns; however, more severe and permanent forms of morbidity including chronic pain, neuropathies, and gait disturbances are also possible, having a lasting impact on patients’ daily activities and quality of life.^3^^,^^4^

To our knowledge, there is no empirical analysis focusing on the range of complications and their prevalence. This lack of comprehensive data limits clinicians’ ability to estimate potential donor-site complications, ultimately affecting the quality of shared decision-making and informed consent.

To address this shortcoming, we performed a systematic review and meta-analysis, focusing on clinically verified objective outcomes (i.e. necrosis) and subjective, patient-reported outcome measures (i.e. pain) associated with free flaps originating from the toes and dorsum of the foot. These specific flaps were chosen as they are the most frequently reported free flaps harvested from the foot. The medial plantar flap, while a valuable resource in reconstructive surgery, is more commonly used as a pedicled flap and involves the removal of specialized plantar tissue which is directly responsible for weight distribution and gait mechanics. Consequently, harvesting this flap creates a fundamentally different means of donor site morbidity, introducing clinically relevant heterogeneity and undermining the validity of the pooled meta-analysis. The primary objective of this analysis was to quantify the incidence of donor-site morbidity in these donor-sites, with a secondary aim to identify the most common complications associated with these flaps.

## Methods

This systematic review and meta-analysis was performed in accordance with the Preferred Reporting Items for Systematic Reviews and Meta-Analyses (PRISMA) checklist (Supplemental Digital Content 1). A systematic search of PubMed, Embase, CENTRAL, Web of Science, and clinicaltrials.gov was performed up to and including March 3, 2025, using MeSH terms and keywords related to: (1) the site of interest (e.g., ‘toe pulp flap,’ ‘free toe flap,’ ‘toe-to-thumb transfer,’ ‘dorsalis pedis flap,’ ‘FDMA flap’); (2) the surgical procedure (e.g., ‘free tissue flap,’ ‘surgical flaps’); (3) donor-site outcomes (e.g., ‘donor site,’ ‘donor-site morbidity’); and (4) postoperative and patient-reported outcomes (e.g., ‘morbidity,’ ‘postoperative complications,’ ‘adverse effects,’ ‘patient-reported outcome measures’) . The complete search Y strategy is provided as supplementary material to this manuscript (Supplemental Digital Content 2).

**Figure 1:**
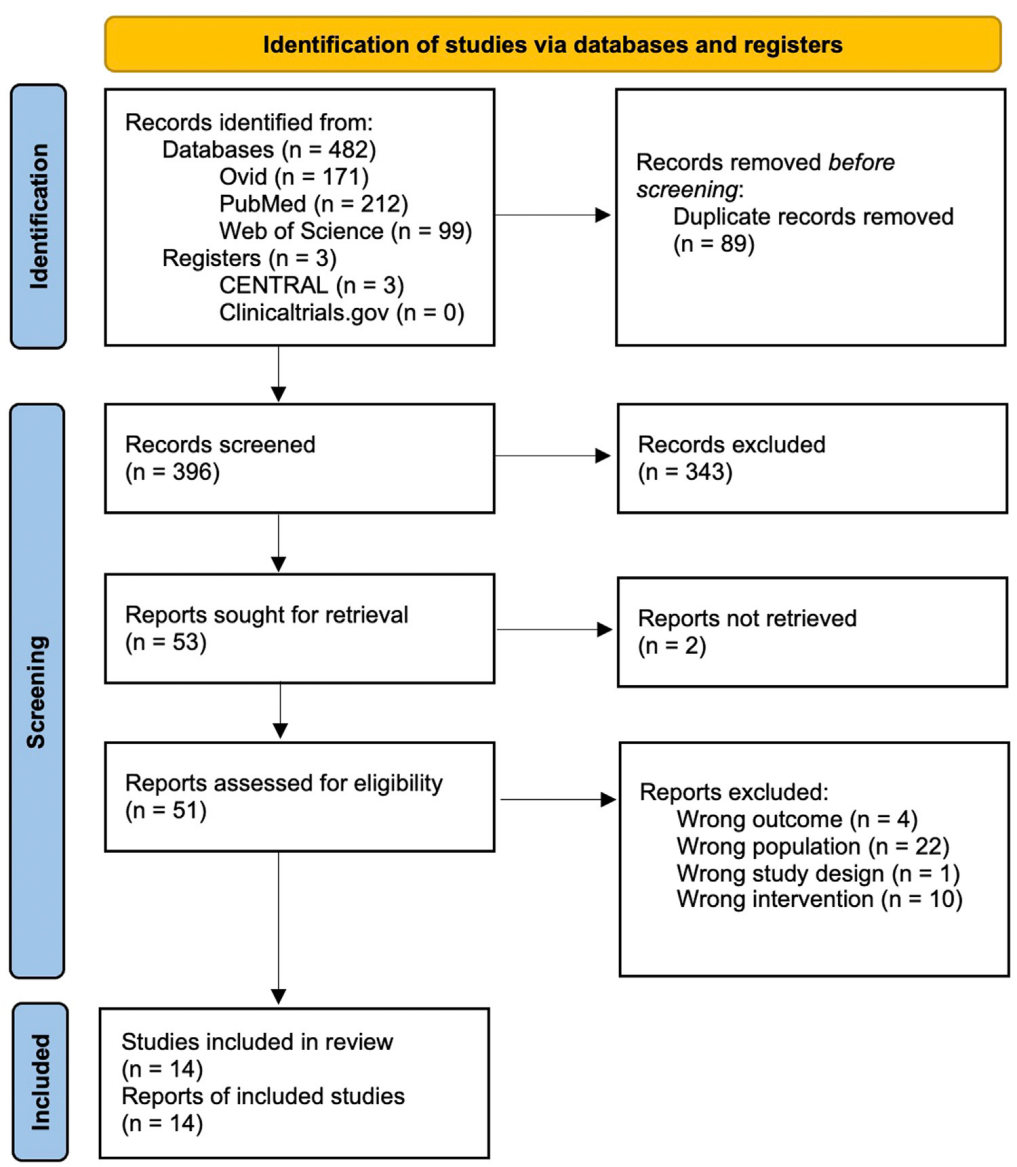
Flow diagram of the study selection process.

Studies of interest were those involving adults (≥18 years) undergoing free flap harvest from the toes or dorsum of the foot, reporting at least one postoperative outcome of the donor-site. Children were excluded as they could inaccurately or insufficiently report subjective complaints. Eligible designs were case series (≥5 patients), observational studies, randomized controlled trials, and controlled clinical trials published in English. The English-language restriction was justified to prevent misinterpretation and unreliable outcome retrieval caused by translation limitations. Letters, reviews, editorials, conference abstracts, animal, and cadaveric studies were excluded. Studies were excluded if they did not have donor-site morbidity as an outcome, if the donor-site could not be evaluated independently, (for example, if the donor-site was covered with a free flap) or when outcome measures were expressed in a way that the proportion of participants experiencing donor-site morbidity could not be calculated. Figure 1 shows the screening and selection processes. Database search results were uploaded into Rayyan.ai,^5^ and duplicate studies were removed. Two reviewers (V.K. & G.D.V.) independently applied the eligibility criteria to identify relevant articles based on titles and abstracts, followed by full-text review. Studies excluded after full-text review are listed in the Appendices (Supplemental Digital Content 3). A third reviewer (L.T.) resolved conflicts. Two reviewers (V.K. and G.D.V.) independently retrieved data from each report and recorded data using an Excel spreadsheet. Discrepancies were resolved by consensus. In the case of missing data, the study investigators were contacted for additional details. The data collection form template and analyzed data can be found as supplementary material (Supplemental Digital Content 4 and 5, respectively). To assess inter-rater reliability for data extraction, both reviewers independently extracted the primary outcome, donor-site morbidity, from all included studies. Agreement between reviewers during full-text screening, data extraction, and risk of bias assessments was assessed using Cohen’s Kappa, calculated with SPSS Version 29. Grey literature and unpublished data were not included nor sought out in this review.

The primary outcome of interest was objective and subjective donor-site morbidity, expressed as the percentage of patients experiencing at least one donor-site complication. Objective morbidity was defined as complications detected on physical examination or via clinical criteria (e.g., infection, hematoma, dehiscence, necrosis, graft loss, re-operation), whereas subjective morbidity included patient-reported symptoms such as sensory impairment, pain, footwear restrictions, poor appearance, and daily life disturbances. An overview of the complications/symptoms and measurement scales utilized by each study can be found in the appendices (Supplemental Digital Content 6).

Two reviewers (V.K.R. & G.D.V.) critically appraised each paper using the Newcastle-Ottawa Scale (NOS) for cohort studies and the Joanna Briggs Institute (JBI) Critical Appraisal Checklist for case series. The JBI checklist was scored as low-, moderate-, or high-risk. For the NOS, the final score of each study was categorized as good, fair, or poor based on the Agency for Healthcare Research and Quality (AHRQ) standards.

Publication bias and small-study effects were assessed using funnel plots for visual asymmetry and Egger’s regression test for statistical confirmation. The trim-and-fill method adjusted pooled estimates if significant bias was detected.

Studies rated as high-risk of bias were not excluded in the primary meta-analysis. Alternatively, to evaluate their influence on pooled morbidity estimates, a structured series of sensitivity and subgroup analyses were also performed, in which: (1) all high-risk studies were excluded, (2) studies with a high risk of detection bias were excluded, and (3) pooled morbidity was compared between low-, moderate-, and high-risk study groups. This allowed us to assess whether risk-of-bias categories acted as moderators and determine whether exclusion of high-risk studies altered the results.

Blinding was expected to be rare in this surgical context; however, studies that utilized standardized, objective measurement tools, patient charts, or independent assessors for donor-site morbidity evaluation were considered at a lower risk of detection bias. Comparing registered protocols with published methods was not possible because the majority of included studies were observational studies or case series, for which previously published protocols or registration is not required and therefore not reported. Consequently, evaluation of selective reporting was limited to internal consistency between the outcomes specified in the methods section and those reported in the results.

Loss to follow-up was evaluated by reviewing reported attrition rates, categorized as high (>20%) or low. Sensitivity analyses would assess its impact by excluding studies with high attrition to test the robustness of pooled estimates.

SPSS and R studio (metafor, meta, and dplyr packages) were used for statistical analysis. The statistical analysis was separated into two separate parts -objective and subjective morbidity- reflecting differences in outcome ascertainment, clinical interpretation, and susceptibility to detection and reporting bias. Objective and subjective outcomes are distinct manifestations of donor-site morbidity and should therefore not be pooled. As the outcome of donor-site morbidity was binary (presence or absence), the Freeman-Tukey double arcsine transformation was applied to the proportions to stabilize variances and allow the inclusion of studies reporting 0% or 100% morbidity without requiring continuity corrections. Effect sizes and variances were calculated using the Freeman-Tukey transformed scale. Data was pooled using a random-effects meta-analysis model to account for variability among studies using the metafor package in R. The pooled Freeman-Tukey estimates were back-transformed to the original proportion scale for interpretability. This approach ensured robust synthesis of morbidity rates across studies with varying sample sizes and event rates. Effect sizes were expressed as percentages with corresponding 95% confidence intervals (CI). Heterogeneity among included studies was evaluated using the I² statistic and Tau-squared (τ^2^), with the following I^2^ thresholds: 0–25% (low heterogeneity), 25–50% (moderate heterogeneity), and >50% (high heterogeneity). As the uncertainty of I^2^ increases with a smaller number of studies, this value was interpreted with caution, especially when assessing fewer than 10 studies. Sensitivity analyses were performed to explore the sources of heterogeneity using leave-one-out analysis. The restricted maximum likelihood (REML) estimator was used to estimate the between-study variance (τ²).

Subgroup analyses were conducted if at least two studies per subgroup provided data on flap type, age, or other relevant factors. The aim of these analyses was to explain heterogeneity observed in the overall meta-analysis, with significant subgroup differences (*p* < 0.05) indicating potential effect modifiers. Pooled proportions were compared using a random-effects model and Cochran’s Q test to evaluate statistical significance.

The protocol for this review can be found on PROSPERO using the following identification number: CRD420250655121.

## Results

### Overview

Initially, 396 studies were screened for eligibility based on titles and abstracts. Of these, the full-text of 53 articles were sought for retrieval; however, two articles could not be located. Both authors were contacted; however, one author did not respond,^6^ and the other denied the article’s existence in English.^7^

The final analysis included 14 studies^3^^,^^8^, ^9^, ^10^, ^11^, ^12^, ^13^, ^14^, ^15^, ^16^, ^17^, ^18^, ^19^, ^20^ comprising 394 cases. Table 1 presents the general characteristics of the studies included in this meta-analysis. The pooled objective donor-site morbidity was 2.99% (95% CI 0.60–7.12%), and the pooled subjective donor-site morbidity was 3.32% (95% CI 1.62–5.60%). Heterogeneity was high for objective morbidity (I² = 88.12%, τ^2^ = 0.1004) and moderate for subjective morbidity (I² = 52.0%, τ² = 0.0181). Cohen’s Kappa for the final inclusion of studies was 0.757 (SE = 0.099, *p* < 0.001), and 0.769 (SE = 0.105, *p* < 0.001) for data extraction, indicating substantial agreement between reviewers.

**Table 1:** General characteristics of included studies.

Study ID	N	Age (Mean)	Sex (M: F)	Start date	Study design	Flap type	Donor closure (N)
Evin et al.^14^	33	36.8	23:10	2018	Cohort	Toe	Primary (33)
Ma et al.^12^	14	35.2	14:0	2011	Case series	Dorsalis pedis	STSG (14)
Li et al.^8^	5	22	3:2	1994	Case series	Toe	Primary (5)
del Piñal et al.^15^	8	33	Unknown	Unknown	Case series	Toe	Unknown
Zhao et al.^9^	6	25.5	4:2	2002	Case series	Toe	Primary (6)
Li et al.^16^	12	38.6	7:5	2019	Case series	Toe	FTSG (12)
Kim et al.^3^	246^a^	41.2	34:7	2009	Case series	Toe	Direct (245) & STSG (1)
Ray, et al.^17^	6	31	6:0	2002	Case series	Toe	Unknown
Do Amaral et al.^19^	8	34.6	8:0	1983	Case series	Dorsalis pedis	Unknown
Gu et al.^18^	21	34.5	13:08	2007	Case series	Toe	Primary (3) & FTSG (18)
Rui et al.^10^	7	32	5:2	2001	Case series	Toe	FTSG (7)
del Piñal et al.^13^	6	37	5:1	1997	Case series	Toe	STSG (6)
Kalfarentzos et al.^20^	7	62	5:2	2009	Case series	Dorsalis pedis	FTSG (7)
Huang et al.^11^	15	39.9	13:2	1998	Cohort	Toe	Direct (6), FTSG (6) & STSG (3)

FTSG, Full-thickness skin graft; STSG, Split-thickness skin graft.

a The study by Kim et al. had originally 246 participants from which objective morbidity outcomes were assessed. The subjective outcomes were assessed in a cohort of 54 participants who agreed to follow-up questioning after 1 year.

### Risk-of-bias assessments

Two cohort studies were assessed using the Newcastle-Ottawa Scale (Table 2). The studies by Evin et al.^14^ and by Huang et al.^11^ were rates as low-risk and high-risk of bias, respectively.

**Table 2:** Risk of bias of included cohort studies according to the Newcastle Ottawa Scale.

	Newcastle Ottawa Scale for cohort studies								
	Section 1: Selection				Section 2: Comparability	Section 3: Outcome			Final assessment
	Representativeness of the exposed cohort	Selection of the non-exposed cohort	Ascertainment of exposure	Demonstration that outcome of interest was not present at start of study	Comparability of cohorts on the basis of the design or analysis	Assessment of outcome	Was follow-up long enough for outcomes to occur	Adequacy of follow up of cohorts	
Evinet al.^14^	**✩**	**✩**	**✩**	**✩**	**✩✩**	**✩**	**✩**	**✩**	**Good**
Huang et al.^11^	**✩**	**✩**	**✩**			**✩**	**✩**	**✩**	**Poor**

Good quality studies required 3–4 stars in the selection domain, 1–2 stars in the comparability domain, and 2–3 stars in the outcome/exposure domain. Fair quality studies required 2 stars in the selection domain, 1–2 stars in the comparability domain, and 2–3 stars in the outcome/exposure domain. Studies were considered poor quality if they achieved 0–1 star in the selection domain, 0 stars in the comparability domain, or 0–1 stars in the outcome/exposure domain.

Twelve case series were assessed using the JBI Checklist (Table 3). Due to the surgical context of the interventions, and inability to standardize patients’ trauma, item 2 of the checklist, ‘Was the condition measured in a standard, reliable way for all participants included in the case series?’ was considered inapplicable and therefore removed. Only one of the included case series by Kim et al.^3^ reported patient comorbidities; consequently, studies were considered to have sufficiently reported on patient demographics if they reported patient age and sex. Based on this modified checklist, three studies were rated low-risk, seven moderate, and two high-risk of bias. Cohen´s Kappa for risk-of-bias assessment agreement was 0.563 (SE = 0.174, *p* = 0.002), indicating moderate agreement between authors.

**Table 3:** Risk of bias of included case series according to the JBI checklist.

JBI checklist for case series	Ma et al.^12^	Li et al.^8^	del Piñal et al.^15^	Zhao et al.^9^	Li et al.^16^	Kim et al.^3^	Ray, et al.^17^	Gu et al.^18^	Rui et al.^10^	del Piñal et al.^13^	Do Amaral et al.^19^	Kalfarentzos et al.^20^
1	Yes	Yes	Yes	Yes	Yes	Yes	Yes	Yes	Yes	Yes	No	Yes
2	Yes	Yes	Yes	Yes	Yes	Yes	Yes	Yes	Yes	Yes	Yes	Yes
3	Yes	Yes	Unclear	Unclear	Unclear	Yes	Yes	Yes	Unclear	Yes	Unclear	Unclear
4	Yes	Yes	Unclear	Unclear	Unclear	Yes	Yes	Yes	Unclear	Unclear	Unclear	Unclear
5	Yes	Yes	No	Yes	Yes	Yes	Yes	Yes	No	Yes	No	No
6	Yes	Yes	Yes	Yes	Yes	Yes	Yes	Yes	Yes	Yes	Yes	Yes
7	No	No	Yes	Yes	Yes	Yes	No	Yes	Yes	Yes	Yes	Yes
8	Yes	Yes	Yes	Yes	Yes	Yes	Yes	Yes	Yes	Yes	No	No
9	Yes	No	Yes	Yes	Yes	Yes	Yes	Yes	Yes	Yes	Yes	Yes
Risk of bias	Moderate	Moderate	Moderate	Moderate	Moderate	Low	Moderate	Low	Moderate	Low	High	High

JBI case series criteria: 1, clear inclusion criteria; 2, valid method for identification of condition for participants; 4, consecutive inclusion; 5, complete inclusion; 6, clear reporting of participant demographics; 7, clear reporting of clinical information; 8, clear reporting of outcomes or follow-up; 9, clear reporting of presenting site/clinic demographics; 10, appropriate statistical analysis.

A score of 1 was given for ‘yes’ responses, −1 for ‘no’, and 0 for ‘unclear’. A percentage of the score out of the total number of applicable items was calculated and categorized as: high-risk (<50%), moderate-risk (50–80%), and low-risk (>80%).

Detection bias ratings per study are presented in Table 4. No study failed to report subjective outcomes which were stated in the methods section; however, three studies, Li et al.,^8^ Zhao et al.,^9^ and Rui et al.,^10^ failed to mention their outcome measures of interest in the methods section of their manuscript. Studies that used patient records or standardized questionnaires/scales/tests and/or independent assessors to detect outcomes were considered low-risk. Studies were considered high-risk if there was a lack of information on detection methods or assessors of outcomes. Cohen’s Kappa for detection bias assessment was 0.601 (SE = 0.13, *p* < 0.001).

**Table 4:** Detection bias assessment of included studies.

Detection bias													
Evin et al.^14^	Huang et al.^11^	Ma et al.^12^	Li et al.^8^	del Piñal et al.^15^	Zhao et al.^9^	Li et al.^16^	Kim et al.^3^	Ray, et al.^17^	Gu et al.^18^	Rui et al.^10^	del Piñal et al.^13^	Do Amaral et al.^19^	Kalfarentzos et al.^20^
Low	Low	High	High	Moderate	High	Low	Low	Moderate	Low	High	Low	High	Low

### Objective morbidity

**Figure 2:**
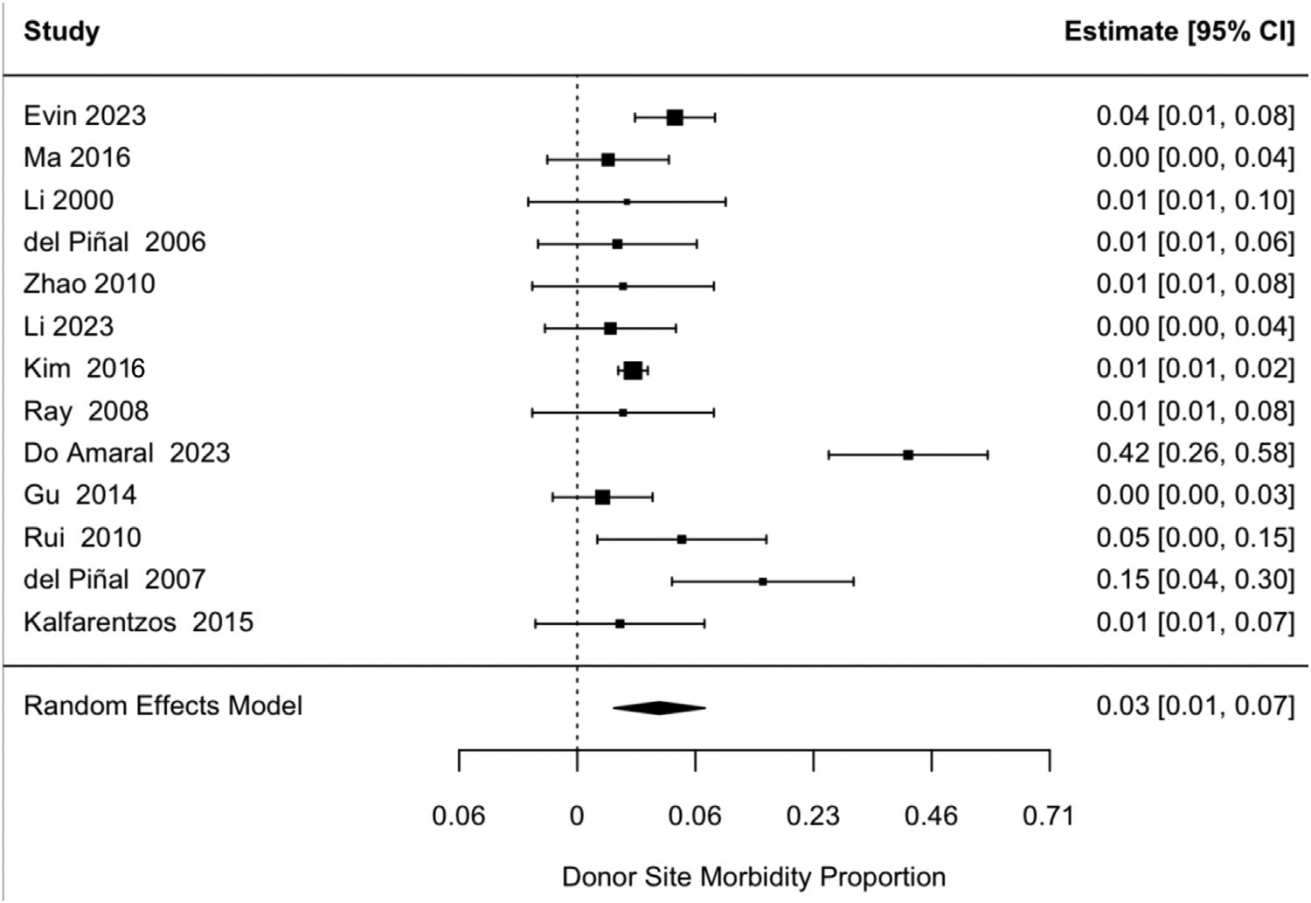
Forest plot of included studies reporting proportions of patients experiencing objective donor site morbidity. Proportions were back-transformed from Freeman-Tukey double arcsine-transformed values and pooled using a random-effects model.

Table 5 shows morbidity outcomes per study. A random-effects meta-analysis using pooled Freeman-Tukey transformed proportion found a morbidity of 0.35 (95% CI 0.16–0.54, *p* = 0.0004). After back-transformation to the original scale, the morbidity was 2.99% (95% CI 0.60–7.12%) (See forest plot in Figure 2). Heterogeneity was significant (Q(12) = 60.38, *p* < 0.001, I² = 88.12%, τ^2^ = 0.1004 (SE = 0.0509).

**Table 5:** Objective morbidity outcomes.

Study ID	N	NM	Objective morbidity (%)	Delayed wound healing	Wound dehiscence	Hematoma	Necrosis	Infection	Graft loss	Second operation
Evin et al.^14^	33	5	15.15		5			1		
Ma et al.^12^	14	0	0							
Li et al.^8^	5	0	0							
del Piñal et al.^15^	8	0	0							
Zhao et al.^9^	6	0	0							
Li et al.^16^	12	0	0							
Kim et al.^3^	246	13	5.28		8	5				
Ray, et al.^17^	6	0	0							
Do Amaral et al.^19^	8	8	100	8						
Gu et al.^18^	21	0	0							
Rui et al.^10^	7	1	14.29	1			1			
del Piñal et al.^13^	6	3	50	3					1	1
Kalfarentzos et al.^20^	7	0	0							
Total	379	30		12	13	5	1	1	1	1
Percentage (%)		7.92		3.17	3.43	1.32	0.26	0.26	0.26	0.26

Objective morbidity was calculated based off raw data.

N, Number of participants in each study; NM, Number of participants who experienced morbidity.

Leave-one-out analysis did not indicate that any of the included studies significantly altered the pooled proportion; however, omitting do Amaral et al.^19^ reduced heterogeneity from high (I² = 88.12%) to low (I² = 32%) (Table 7).

**Figure 3:**
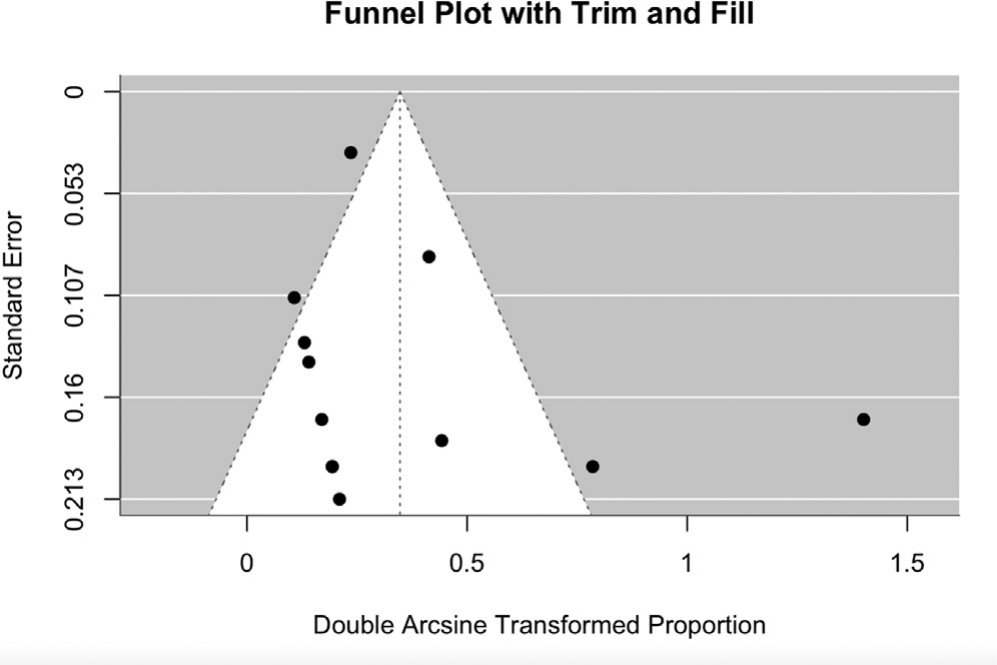
Funnel plot of included studies reporting objective morbidity outcomes, using Freeman-Tukey transformed proportions with Trim and Fill correction.

Visual inspection of the funnel plot (Figure 3) did not demonstrate a pattern consistent with small-study effects, despite the presence of plausible outliers such as do Amaral et al.^19^ The funnel plot was relatively symmetric, and no studies fell outside the expected range. Additionally, the trim-and-fill method did not impute any missing studies or alter the pooled estimate. This was confirmed with Egger's regression test (*z* = 0.61, *p* = 0.54; intercept=0.17 (95% CI: −0.42 to 0.77).

Given the substantial heterogeneity observed, additional analyses were performed to identify possible clinical and methodological sources.

Meta-regression excluding high-risk studies according to the JBI Checklist or NOS (*n* = 3) produced a pooled morbidity of 1.50% (95% CI: 0.73–2.54%). The moderator coefficient was −0.37 (*p* = 0.063), suggesting a non-statistically significant trend of lower morbidity in low-risk studies. A subgroup meta-analysis showed that the pooled morbidity was highest among high-risk studies, estimated at 9.20% (95% CI: 0.02–32.21%). Moderate-risk studies reported a lower pooled morbidity of 0.83% (95% CI: 0.08–2.36%), and low-risk studies reported a morbidity of 2.07% (95% CI: 0.59–4.42%). However, a mixed-effects meta-regression found no significant differences in morbidity between bias groups (QM(2) = 3.32, *p* = 0.19, τ^2^ = 0.0906 (SE = 0.0518)).

A sensitivity meta-analysis was conducted, excluding high-risk studies in any domain (*n* = 8). The pooled morbidity was 1.19% (95% CI: 0.63–1.93%), with high residual heterogeneity across remaining studies (I² = 83.32%, *p* < 0.001, τ^2^ = 0.0866 (SE = 0.0474)). Meta-regression analysis revealed no statistically significant difference (*p* = 0.12).

A meta-analysis excluding studies with a high risk of detection bias (*n* = 6) produced a pooled morbidity of 1.62% (95% CI: 0.43–3.56%). Meta-regression showed no statistically significant difference (QM(1) = 0.44, *p* = 0.51), and substantial heterogeneity remained (I² = 86.5%). Subgroup meta-analysis showed that pooled morbidity estimates were highest in high-risk studies (4.36%, 95% CI: 0.33–12.63%), followed by moderate-risk (2.54%, 95% CI: 1.27–17.60%) and low-risk studies (1.90%, 95% CI: 0.06–8.81%). However, these differences were not statistically significant (QM(2) = 3.32, *p* = 0.19).

Given the high heterogeneity observed in the objective morbidity estimates, subgroup and meta-regression analyses were performed to determine potential explanations. To do this, variations in flap type, closure method, study type, and patient sex and age were examined, however, none of these factors explained a significant proportion of heterogeneity, indicating that they contributed minimally to between-study variance. A study type (cohort or case series) subgroup analysis could not be conducted because there was only one cohort study.

A mixed-effects meta-regression estimated a pooled morbidity of 7.62% (95% CI: 0.66–21.16%) for dorsalis pedis flaps (*n* = 3) and 2.01% (95% CI: 0.12–6.13%) for toe flaps (*n* = 10), with no statistically significant difference (β = −0.27, *p* = 0.23).

In contrast, a mixed-effects meta-regression model showed a significant effect of trial start period on morbidity (QM(3) = 38.11, *p* < 0.0001), explaining 94.5% of the between-study heterogeneity (R² = 94.48%). Estimated morbidity rates decreased substantially over time, with a morbidity estimate of 41.6% (95% CI: 24.3–59.9%) for 1980–1989, 6.6% (95% CI: 1.2–16.0%) for 1990–2000, 1.2% (95% CI: 0.3–2.7%) for 2001–2010, and 1.8% (95% CI: 0.3–4.5%) for 2011–2020.

### Subjective morbidity

**Figure 4:**
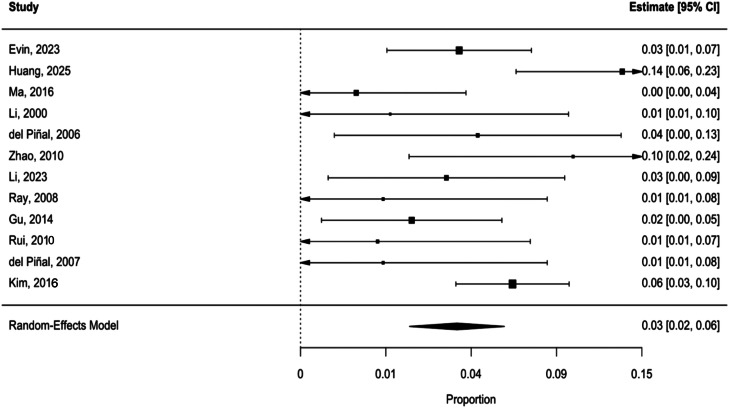
Forest plot of included studies reporting proportions of patients experiencing subjective donor site morbidity. Proportions were back-transformed from Freeman-Tukey double arcsine-transformed values and pooled using a random-effects model.

Subjective morbidity outcomes per study are presented in Table 6, predominantly consisting of functional limitations, pain, poor appearance, and sensory disturbances. Using a random-effects model with REML estimation, the pooled Freeman–Tukey transformed proportion was 0.37 (95% CI: 0.26–0.48, *p* < 0.0001). Between-study heterogeneity was high (*Q*(11) = 21.86, *p* = 0.025, I² = 52.0%, τ² = 0.0181 (SE = 0.0159)). The back-transformed proportions showed that the percentage of patients experiencing morbidity was 3.32% (95% CI 1.62–5.60%) (see forest plot in Figure 4).

**Table 6:** Subjective morbidity outcomes.

Study ID	N	NM	Subjective morbidity (%)	Pain/Metatarsalgia, Hyperalgesia	Hypersensitivity	Numbness/Diminished sensation	Cold intolerance	Daily life/Work disturbance	Physical activity disturbance	Poor appearance
Evin et al.^14^	33	4	12.12	1			3			
Huang et al.^11^	15	7	4.67	2	3	2				
Ma et al.^12^	14	0	0							
Li et al.^8^	5	0	0							
del Piñal et al.^15^	8	1	12.50	1						
Zhao et al.^9^	6	2	33.33	1						1
Li et al.^16^	12	1	8.33							1
Kim et al.^3^	54	12	22.22			3		6		3
Ray, et al.^17^	6	0	0							
Gu et al.^18^	21	1	4.76						1	
Rui et al.^10^	7	0	0							
del Piñal et al.^13^	6	0	0							
Total	187	28		5	3	5	3	6	1	5
Percentage (%)		16.28		2.67	1.60	2.67	1.60	3.21	0.53	2.67

Percentages of morbidity were calculated using raw data.

N, Number of participants in each study; NM, Number of participants who experienced morbidity.

**Table 7:** Proportions and heterogeneity of included studies after leave-one-out meta-analysis of objective morbidity outcomes.

Omitted study	Pooled proportion	Lower 95% CI	Upper 95% CI	I²
Evin et al.^14^	0.017	0011	0.024	0.81
Ma et al.^12^	0.019	0.013	0.027	0.81
Li et al.^8^	0.018	0.012	0.026	0.82
del Piñal et al.^15^	0.019	0.012	0.026	0.82
Zhao et al.^9^	0.019	0.012	0.026	0.82
Li et al.^16^	0.019	0.013	0.026	0.82
Kim et al.^3^	0.028	0.016	0.043	0.81
Ray, et al.^17^	0.019	0.012	0.026	0.82
Do Amaral et al.^19^	0.015	0.010	0.022	0.32
Gu et al.^18^	0.020	0.013	0.027	0.81
Rui et al.^10^	0.018	0.012	0.025	0.82
del Piñal et al.^13^	0.017	0.011	0.024	0.79
Kalfarentzos et al.^20^	0.019	0.012	0.026	0.82

Although leave-one-out analysis did not indicate that any of the included studies significantly altered the pooled proportion, omitting Huang et al.^11^ and Ma et al.^12^ reduced the heterogeneity from high (I² = 52%) to low (I² = 23%) and moderate (I² = 42%), respectively (Table 8).

**Table 8:** Proportions and heterogeneity of included studies after leave-one-out meta-analysis of subjective morbidity outcomes.

Omitted study	Pooled proportion	Lower 95% CI	Upper 95% CI	I²
Evin et al.^14^	0.039	0.025	0.055	0.54
Huang et al.^11^	0.032	0.020	0.046	0.23
Ma et al.^12^	0.042	0.029	0.058	0.43
Li et al.^8^	0.039	0.026	0.054	0.53
del Piñal et al.^15^	0.038	0.025	0.053	0.54
Zhao et al.^9^	0.036	0.024	0.051	0.51
Li et al.^16^	0.039	0.026	0.054	0.54
Kim et al.^3^	0.031	0.018	0.046	0.46
Ray, et al.^17^	0.039	0.027	0.054	0.52
Gu et al.^18^	0.041	0.028	0.057	0.50
Rui et al.^10^	0.040	0.027	0.055	0.51
del Piñal et al.^13^	0.039	0.018	0.054	0.52

Pooled proportions have been back-transformed from Freeman–Tukey transformed proportions.

**Figure 5:**
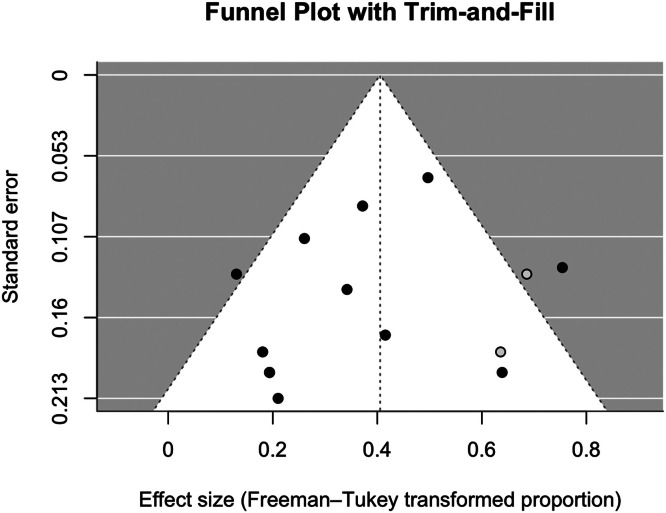
Funnel plot of included studies reporting subjective morbidity outcomes, using Freeman-Tukey transformed proportions with Trim and Fill correction.

The funnel plot of Freeman–Tukey transformed proportions appeared roughly symmetric (Figure 5), with most points falling within the non-significant contour. Application of the trim-and-fill method imputed two missing studies, both with higher morbidity rates; however, the adjusted estimate (4.05%, 95% CI: 2.17–6.49%) remained close to the original (3.32%, 95% CI 1.62–5.60%). Egger’s regression test did not show statistical significance (*z* = –0.93, *p* = 0.35), suggesting no relevant small-study effects.

A sensitivity meta-analysis was performed after excluding high-risk studies (*n* = 4) according to the JBI Checklist or NOS. The pooled morbidity was 3.37% (95% CI: 1.79–5.43%), which was similar to the estimate from the full dataset (3.32%, 95% CI 1.62–5.60%). Meta-regression confirmed that there was no statistically significant difference (*p* = 0.76). A subgroup meta-regression produced pooled morbidity estimates of 3.63% (95% CI: 0.20–11.05%) for the high-risk group, 2.37% (95% CI: 0.77–4.83%) for the moderate-risk group, and 4.31% (95% CI: 2.08–7.28%) for the low-risk group. Meta-regression found no statistically significant differences between the subgroups (QM(2) = 0.31, *p* = 0.85).

A meta-analysis excluding studies with a high risk of bias on either the JBI, NOS, or detection bias assessment (*n* = 6) showed a pooled morbidity estimate of 3.29% (95% CI: 1.14–6.49%). Meta-regression concluded no statistically significant difference (QM(1) = 0.04, *p* = 0.85).

Excluding high-risk studies in the detection bias domain (*n* = 4) yielded a pooled morbidity estimate of 4.02% (95% CI 1.97–6.74%); however, meta-regression found no statistically significant difference (*p* = 0.28).

Subgroup analysis yielded pooled morbidity estimates of 1.81% (95% CI: 0.06–5.92%) in the high-risk group, 2.53% (95% CI 0.11–7.97%) in the moderate-risk group, and 4.32% (95% CI: 1.88–7.72%) in the low-risk group. Meta-regression indicated that these differences were not statistically significant (QM(2) = 1.45, *p* = 0.48).

Subgroup and meta-regression analyses were performed to examine potential moderators of heterogeneity. Start year, study types, and patient sex and age were examined, however, none of these factors explained a significant proportion of heterogeneity, indicating that they contributed minimally to between-study variance. Subjective morbidity gradually declined with more recent studies, although this difference was not statistically significant. A flap-type subgroup analysis could not be conducted because only one study reported dorsalis pedis flap harvest.

Six studies reported only one donor-site closure method: primary (*n* = 3), split-thickness skin graft (*n* = 1), and full-thickness skin graft (*n* = 2). A mixed-effects meta-regression subgroup analysis found a pooled morbidity of 10.34% (95% CI: 1.62–23.13%) in the primary closure group and 2.21% (95% CI: 0.00–15.19%) in the skin graft closure group, but this was not statistically significant (QM(1) = 1.14, *p* = 0.2857). The overall model showed no residual heterogeneity (τ² = 0; I² = 0.00%), although interpretation of this result is limited by the small number of studies.

## Discussion

The meta-analysis consisting of 14 studies and 394 cases found a low pooled incidence of donor-site morbidity after free flap harvest from the foot, estimated at 2.99% (95% CI 0.60–7.12%) for objective outcomes, with wound dehiscence, delayed healing, and hematoma being the most common complications (Table 5). The studies included reported morbidity rates ranging from 0 to 100%, explaining the significant heterogeneity among studies (I² = 88.12%). Subjective morbidity outcomes occurred in 3.32% (95% CI 1.62–5.60%) of patients, with daily life disturbances, pain, numbness, and hypersensitivity occurring most frequently (Table 6). The included studies reported a subjective donor-site morbidity of 0–33.33%, indicating considerable variation between studies. This variability could be due to varying definitions of morbidity, detection methods, and surgical methods. For example, in the study by Kim et al.,^3^ many patients reported pain, but we only considered this morbidity if the pain was at least ‘somewhat limiting.’

The JBI Checklist and NOS risk of bias assessments showed clear variability in the quality of studies. Most studies reported a low or moderate risk of bias in all domains, except detection bias, with multiple studies lacking clarity in their methodology, not utilizing standardized detection methods, or failing to report them at all. Nevertheless, sensitivity analysis excluding high-risk studies did not substantially change our results for both types of morbidity, and meta-regression showed a non-statistically significant difference in pooled estimates across bias levels, supporting the strength of our pooled estimates.

For the subjective morbidity outcomes, application of the trim-and-fill method imputed two missing studies (Figure 5). Nevertheless, Egger’s regression test did not show statistical significance (*z* = –0.93, *p* = 0.35), and the adjusted pooled estimate (4.05%, 95% CI 2.17–6.49%) remained close to the original estimate, suggesting that any publication bias had minimal impact on our estimate of subjective morbidity.

Visual inspection of the funnel plot (Figure 3) of objective morbidity outcomes identifies outliers, such as Do Amaral et al.^19^ and del Piñal et al.,^13^ reporting higher morbidity rates. This morbidity was caused by delayed wound healing in 100% and 50% of patients, respectively. Egger’s regression test was non-significant, further indicating the absence of small-study effects. Additionally, the trim-and-fill method did not impute any missing studies or alter the pooled estimate. These findings suggest no significant publication bias or small-study effects in the included studies.

With regard to attrition bias, no study reported a loss to follow-up, except for Kim et al.,^3^ where a large proportion of patients did not answer follow-up questionnaires after 1 year of follow-up which related to subjective morbidity outcomes. The reported follow-up periods of the studies ranged from 6 months to 10 years, although the study by Kalfarentzos et al.^20^ did not report a follow-up duration.

Only one of the subgroup analyses conducted showed a significant moderator of (objective) morbidity: trial start year. This analysis found a statistically significant decline in objective morbidity rates after the year 2000 while explaining nearly all observed heterogeneity. This trend likely reflects advancements in surgical techniques and perioperative care over recent decades. Flap type, study design (prospective vs. retrospective), sex, age, and closure method did not significantly influence morbidity. This highlights the lack of consistent, statistically significant moderators of donor-site morbidity, suggesting that the variability between studies may be caused by unmeasured or study-specific factors.

This meta-analysis provides novel insights into donor-site morbidity in free flaps from the foot, with the pooled estimate being supported by robust statistical methods. Nevertheless, this study has some limitations, and therefore we estimate the certainty of evidence as moderate. Firstly, this meta-analysis is composed of a small number of studies and participants, in which donor-site morbidity was often not the primary outcome, limiting the generalizability of findings. Restricting inclusion to English-language publications may have introduced language bias, while excluding unpublished data and gray literature could have contributed to publication bias. Heterogeneity between studies is considerably high; to improve this, future studies should standardize outcome detection methods and report on key factors, such as patient comorbidities and standardized outcome detection methods. Variability in the definition and assessment of objective outcome measures, as well as variations in flap types and techniques, also likely contributed to high heterogeneity. Definitions in donor-site morbidity should be better established. In this study, ‘graft loss’ was not considered donor-site morbidity if the wound healed without further intervention.

Additionally, to more accurately assess the effect of flap harvest from the foot, the donor-site should be controlled for sensory and functional impairments preoperatively.

Furthermore, due to a lack of reporting on individual participants, participants were assumed to not have experienced two different types of morbidity unless individually specified. This only applied to subjective complaints reported in two studies: Kim et al.^3^ and Huang et al.^11^ The authors were contacted for further clarification, but no resolution was found. Therefore, potential overlap between complication types could not be ruled out, leading to a possible overestimation of the proportion of participants who experienced subjective donor-site morbidity. As this review excluded children, these results cannot be extrapolated to the pediatric population.

## Conclusion

This meta-analysis showed a pooled donor-site morbidity of 2.99% and 3.32% for objective and subjective outcomes respectively, following free flap harvest from the toes or dorsum of the foot. Sensitivity analysis excluding studies with a high risk of bias produced similar results. Flap type, study design, patient age or sex, and closure method did not significantly affect morbidity; however, objective morbidity was significantly lower in studies with a trial start date after the year 2000, accounting for 94.5% of heterogeneity. These results suggest that the occurrence of donor-site morbidity is relatively low, although substantial heterogeneity and variability in studies limit the strength of these results. Future studies should utilize standardized outcome measures and detailed reporting of patient comorbidities.

## Conflict of interest

None.

## Appendix Supplementary materials

Image, application 1

Image, application 2

Image, application 3

Image, application 4

Image, application 5

Image, application 6
