# Supplementary material for: A systematic review and meta-analysis of the morbidity of the donor-site of flaps harvested based on the first intermetatarsal artery
Source: JPRAS Open. 2026 Jan 17;48:884–901. doi: 10.1016/j.jpra.2026.01.009 (PMC12924899; doi:10.1016/j.jpra.2026.01.009)
Supplement: Supplementary file 2 [file mmc2.docx]

**A Systematic Review and Meta-analysis of the Morbidity of the Donor-Site in Free Flaps Harvested from the Foot: Search Strategy**

**Search Strategy:**

Embase (Ovid), CENTRAL, PubMed, Web of Science, ClinicalTrials.gov, and Cochrane databases will be searched on 03-03-2025, without updates. Restrictions: Articles not written in the English language. No other search filters were used. Included study designs are case series with five or more patients, retrospective and prospective observational studies, randomized controlled-trials, and controlled clinical trials. Letters to the editors, summaries, case reports, review papers, editorials, or unpublished conference abstracts will be excluded. Reference lists will not be examined for potential study inclusion. In the case of missing data, study investigators will be contacted for additional details. Database search results will be uploaded into Rayyan.ai, and duplicate studies will be removed.

**Final Search:** ((((Toe pulp flap) OR (Toe) OR (Free toe) OR (Free toe pulp) OR (Toe transfer) OR (Toe-to-thumb) OR (Dorsal pedis flap) OR (Dorsalis pedis flap) OR (Dorsal metatarsal artery flap) OR (First Dorsal metatarsal artery flap) OR (FDMA Flap)) AND ((Free Tissue Flap) OR (Surgical Flaps))) AND (Outcomes OR Morbidity OR Postoperative Complications OR Patient Reported Outcome Measures OR Adverse effects)) AND (Donor site)

**Study selection:** Two reviewers (V.K.R. & G.D.V.) will independently apply the eligibility criteria to select studies for inclusion in the systematic review. Disagreements will be resolved by a third reviewer (L.T.). Subsequently, blinded full text review was completed by authors (V.K.R. and G.D.V.) and papers meeting full criteria for inclusion will be finalized

Table 1: Search terms

| **Concept** | | | |
| --- | --- | --- | --- |
| **Donor site** | **Surgical procedure** | **Outcome** | **Site of interest** |
| (Toe pulp flap) OR (Toe) OR (Free toe) OR (Free toe pulp) OR (Toe transfer) OR (Toe-to-thumb) OR (Dorsal pedis flap) OR (Dorsalis pedis flap) OR (Dorsal metatarsal artery flap) OR (First Dorsal metatarsal artery flap) OR (FDMA Flap) | Free Tissue Flap OR Surgical Flaps | Morbidity OR Postoperative Complications OR Patient Reported Outcome Measures OR Adverse effects OR Outcomes | Donor site |

.
