# Supplementary material for: A systematic review and meta-analysis of the morbidity of the donor-site of flaps harvested based on the first intermetatarsal artery
Source: JPRAS Open. 2026 Jan 17;48:884–901. doi: 10.1016/j.jpra.2026.01.009 (PMC12924899; doi:10.1016/j.jpra.2026.01.009)
Supplement: Supplementary file 3 [file mmc3.docx]

**Appendices**

Table 1: Studies excluded after full-text review with reasons

| **Reference Number** | **First Author (Year)** | **Reason for Exclusion** |
| --- | --- | --- |
| 1 | Adani (1999) | Wrong population: Children |
| 2 | Adani (2005) | Wrong population: Children |
| 3 | Wang (2021) | Wrong population: Children |
| 4 | Coleman (1985) | Wrong population: Children |
| 5 | Del Piñal (2014) | Wrong population: Children |
| 6 | Ghareeb (2002) | Wrong intervention: Not a free flap |
| 7 | Georgescu (2019) | Wrong population: Children |
| 8 | Stupka (2004) | Wrong population: Could include children |
| 9 | Inoue (1991) | Wrong population: Children |
| 10 | Ju (2019) | Wrong outcome |
| 11 | Chung (2000) | Wrong outcome |
| 12 | Kawamura (2005) | Wrong intervention: Donor site covered with a flap |
| 13 | Kempny (2013) | Wrong population: Children |
| 14 | Lee (1995) | Wrong population: Children |
| 15 | Lee (1994) | Wrong population: Children |
| 16 | Ling (2022) | Wrong outcome |
| 17 | Liu (2012) | Wrong intervention: Wrong flap type |
| 18 | Lowdon (1987) | Wrong population: Children |
| 19 | Samson (1998) | Wrong intervention: Not a free flap |
| 20 | Ratcliffe (1991) | Wrong population: Children |
| 21 | Woo (2006) | Wrong population: Children |
| 22 | Shen (2016) | Wrong population: Children |
| 23 | Sun (2016) | Wrong population: Children |
| 24 | Chu (2022) | Wrong population: Children |
| 25 | Matsusue (2024) | Wrong intervention: Donor site covered with a flap |
| 26 | Tsai (2019) | Wrong outcome |
| 27 | Tang (2022) | Wrong intervention: Donor site covered with a flap |
| 28 | Wang (2023) | Wrong intervention: Not a free flap |
| 29 | Woo (2004) | Wrong intervention: Not a free flap |
| 30 | Fuse (2022) | Wrong population: Children |
| 31 | Zhang (2019) | Wrong population: Children |
| 32 | Zhang (2022) | Wrong population: Children |
| 33 | Zhang (2015) | Wrong population: Children |
| 34 | Dong (2003) | Wrong intervention: Donor site covered with a flap |
| 35 | Duncan (1985) | Wrong population: Children |
| 36 | Del Piñal (2005) | Wrong study design: Case series <5 |
| 37 | Wang (2019) | Wrong intervention: Donor site covered with a flap |

References of studies excluded after full-text review

1. Adani R, Cardon LJ, Castagnetti C, et al. Distal thumb reconstruction using a mini wrap-around flap from the great toe. 1999.

2. Adani R, Marcoccio I, Tarallo L, et al. The aesthetic mini wrap-around technique for thumb reconstruction. 2005.

3. Wang C, Yang W, Zhang F, et al. Superficial peroneal neurocutaneous flap for coverage of donor site defect after the combined transfer of toe and dorsal foot flap. 2021.

4. Coleman DA, Urbaniak JR. Osteocutaneous flaps for thumb and digit reconstruction. In unique situations. 1985.

5. del Piñal F, Moraleda E, de Piero GH, et al. Onycho-osteo-cutaneous defects of the thumb reconstructed by partial hallux transfer. 2014.

6. Ghareeb FM. Using the extensor digitorum brevis muscle to improve donor-site morbidity of the dorsalis pedis flap. 2002.

7. Georgescu AV, Battiston B, Matei IR, et al. Emergency toe-to-hand transfer for post-traumatic finger reconstruction: A multicenter case series. 2019.

8. Stupka I, Vesely J, Drazan L, et al. Foot morbidity following toe to hand transfers. 2004.

9. Inoue G, Maeda N, Suzuki K. Closure of big toe defects after wrap-around flap transfer using the arterialized venous flap. 1991.

10. Ju J, Li L, Hou R. Transplantation of a free vascularized joint flap from the second toe for the acute reconstruction of defects in the thumb and other fingers. 2019.

11. Chung KC, Wei FC. An outcome study of thumb reconstruction using microvascular toe transfer. 2000.

12. Kawamura K, Yajima H, Kobata Y, et al. Coverage of big toe defects after wrap-around flap transfer with a free soleus perforator flap. 2005.

13. Kempny T, Paroulek J, Marik V, et al. Further developments in the twisted-toe technique for isolated thumb reconstruction: our method of choice. 2013.

14. Lee KS, Chae IJ, Hahn SB. Thumb reconstruction with a free neurovascular wrap-around flap from the big toe: long-term follow-up of thirty cases. 1995.

15. Lee KS, Park SW, Kim HY. Tendocutaneous free flap transfer from the dorsum of the foot. 1994.

16. Ling L, Li X. A dorsalis pedis venous flap containing a U-shaped venous arch for the reconstruction of fingertip defects. 2022.

17. Liu F, Dong J, Wang T, et al. Reconstruction of through-and-through facial defects with combined anterior tibial flap and dorsalis pedis flap. 2012.

18. Lowdon IM, Nunley JA, Goldner RD, et al. The wraparound procedure for thumb and finger reconstruction. 1987.

19. Samson MC, Morris SF, Tweed AEJ. Dorsalis pedis flap donor site: Acceptable or not? Plastic and Reconstructive Surgery. 1998.

20. Ratcliffe RJ, McGrouther DA. Free toe pulp transfer in thumb reconstruction. Experience in the West of Scotland Regional Plastic Surgery Unit. 1991.

21. Woo SH, Lee GJ, Kim KC, et al. Cosmetic reconstruction of distal finger absence with partial second toe transfer. 2006.

22. Shen XF, Mi JY, Xue MY, et al. Modified great toe wraparound flap with preservation of plantar triangular flap for reconstruction of degloving injuries of the thumb and fingers: Long-term follow-up. 2016

23. Sun W, Chen C, Wang Z, et al. Full-length finger reconstruction for proximal amputation with expanded wraparound great toe flap and vascularized second toe joint. 2016.

24. Chu T, Xiao J, Tao Z, et al. A hitchhiking approach to reconstruct the finger pulp and the subsequent 1st toe hemi-pulp donor site defect. 2022.

25. Matsusue T. Nail reconstruction using an osteo-onychocutaneous flap with the dorsal digital artery of the hallux as the pedicle: Techniques and anatomical insights. 2024.

26. Tsai TY, Fries CA, Hsiao JC, et al. Patient-reported outcome measures for toe-to-hand transfer: A prospective longitudinal study. 2019.

27. Tang L, Zhao X, Zou Y. Combined great toe dorsal nail-skin flap and medial plantar flap for one-stage reconstruction of degloved finger. 2022.

28. Wang J, Xue M, Lu H, et al. Functional and aesthetic recovery of the second toe defect using a wrap-around pedicled flap from the great toe. 2023.

29. Woo S, Kim J, Seul J. Immediate toe-to-hand transfer in acute hand injuries: Overall results, compared with results for elective cases. 2004.

30. Fuse Y, Yamamoto T, Kageyama T, et al. Domino free flap transfer using a superficial circumflex iliac artery perforator flap for the toe flap donor site. 2022.

31. Zhang G, Ju J, Li L, et al. Combination free foot flaps for digit reconstruction: A retrospective analysis of 37 cases. 2019.

32. Zhang X, Wang Z, Ma X, et al. Repair of finger pulp defects using a free second toe pulp flap anastomosed with the palmar vein. 2022.

33. Zhang X, Chen C, Li Y, et al. Does nerve repair influence the outcome of reconstruction of a digital nail defect using a free composite flap taken from the great toe? 2015.

34. Dong JS, Peng YP, Zhang YX, et al. Reverse anterior tibial artery flap for reconstruction of foot donor site. 2003.

35. Duncan MJ, Zuker RM, Manktelow RT. Resurfacing weight bearing areas of the heel. The role of the dorsalis pedis innervated free tissue transfer. 1985.

36. Del Piñal F, Garcia-Bernal FJ, Delgado J, et al. Overcoming soft-tissue deficiency in toe-to-hand transfer using a dorsalis pedis fasciosubcutaneous toe free flap: Surgical technique. 2005.

37. Wang Z, Sun W, Lineaweaver WC, et al. Distal finger reconstruction by bilateral lateral hallux osteo-onychocutaneous free flap. 2019.
