# Supplementary material for: A systematic review and meta-analysis of the morbidity of the donor-site of flaps harvested based on the first intermetatarsal artery
Source: JPRAS Open. 2026 Jan 17;48:884–901. doi: 10.1016/j.jpra.2026.01.009 (PMC12924899; doi:10.1016/j.jpra.2026.01.009)
Supplement: Supplementary file 6 [file mmc6.docx]

# Complications/Symptoms and Measurement Scales Across Studies

| Study | Objective Morbidity | Subjective Morbidity |
| --- | --- | --- |
| Evin, 2023 | Not specified | Limitations – FFI; Sensory recovery – SWM, s2PD |
| Huang, 2025 | NA | Sensibility – s2PD, m2PD, SWM;  FADI; Discomfort – direct questioning; Scars – VSS; |
| Ma, 2016 | Not specified | Sensory testing – Pain perception, temperature, vibratory threshold, s2PD |
| Li, 2000 | Not specified | Not specified |
| Del Piñal, 2006 | Not specified | Foot complaints – questioning; Cosmetic – VAS; AOFAS lesser toes |
| Zhao, 2010 | Not specified | Appearance, function, pain – MHQ |
| Li, 2023 | Not specified | Scars – Modified VSS; Sensory recovery– s2PD |
| Kim, 2016 | Patient records (Infection, necrosis, dehiscence, hematoma) | Pain, meds, appearance, gait disturbance, daily limitations – questionnaires; Sensory recovery – SWM; Nail deformities – patient records |
| Ray, 2008 | Patient records | Patient records |
| Do Amaral, 2023 | Not specified | NA |
| Gu, 2014 | Assessed by senior surgeon | Sensitivity – s2PD, SWM; Cold intolerance – CISS; Pain/aesthetics – VAS; Foot pathology – FFI |
| Rui, 2010 | Clinical observation (color, swelling, capillary refill, temperature) | Not specified |
| Del Piñal, 2007 | Patient records | Cosmetic – VAS; Functional limitation – AOFAS lesser toes |
| Kalfarentzos, 2015 | Patient records | NA |

**Abbreviations:**

FFI = Foot function Index

SWM = Semmes–Weinstein monofilament test

s2PD = Static two-point discrimination

m2PD = Moving two-point discrimination

FADI = Foot and Ankle Disability Index

VSS = Vancouver Scar Scale

VAS = Visual analogue scale

AOFAS = American Orthopaedic Foot and Ankle Society

MHQ = Michigan Health Questionnaire

CISS = Cold Intolerance Severity Score
